# 7-Chloro-3,3-dimethyl-9-phenyl-1,2,3,4-tetra­hydro­acridin-1-one

**DOI:** 10.1107/S1600536809050326

**Published:** 2009-11-28

**Authors:** Wan-Sin Loh, Hoong-Kun Fun, S. Sarveswari, V. Vijayakumar, B. Palakshi Reddy

**Affiliations:** a X-ray Crystallography Unit, School of Physics, Universiti Sains Malaysia, 11800 USM, Penang, Malaysia; b Organic Chemistry Division, School of Advanced Sciences, VIT University, Vellore 632 014, India

## Abstract

In the title salt, C_21_H_18_ClNO, the quinoline ring system is approximately planar [maximum deviation = 0.035 (2) Å], and forms a dihedral angle of 71.42 (6)° with the attached phenyl ring. The cyclo­hexa­none ring exists in a half-boat conformation. In the crystal packing, C—H⋯O contacts link the mol­ecules into extended supra­molecular chains along the *c* axis.

## Related literature

For background to and biological activity of quinolines, see: Morimoto *et al.* (1991 ▶); Michael (1997 ▶); Markees *et al.* (1970 ▶); Campbell *et al.* (1988 ▶); Maguire *et al.* (1994 ▶); Kalluraya & Sreenivasa (1998 ▶); Roma *et al.* (2000 ▶); Chen *et al.* (2001 ▶). For the synthesis of quinoline derivatives, see: Fun, Loh *et al.* (2009 ▶); Fun, Yeap *et al.* (2009 ▶). For a related structure: see: Loh *et al.* (2009 ▶). For ring conformations, see: Cremer & Pople (1975 ▶). For the stability of the temperature controller used for the data collection, see: Cosier & Glazer (1986 ▶).
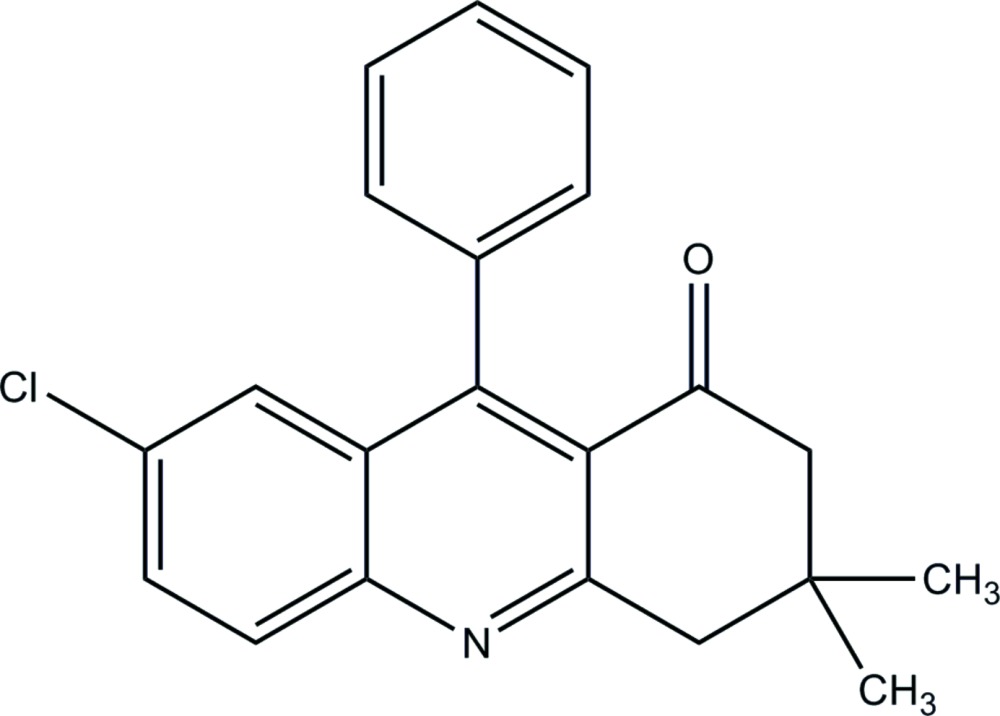


## Experimental

### 

#### Crystal data

C_21_H_18_ClNO
*M* *_r_* = 335.81
Triclinic, 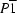

*a* = 9.8375 (1) Å
*b* = 10.0525 (1) Å
*c* = 10.1076 (1) Å
α = 79.162 (1)°
β = 63.389 (1)°
γ = 70.928 (1)°
*V* = 843.59 (2) Å^3^
*Z* = 2
Mo *K*α radiation
μ = 0.23 mm^−1^
*T* = 100 K
0.30 × 0.20 × 0.15 mm

#### Data collection

Bruker SMART APEXII CCD area-detector diffractometer
Absorption correction: multi-scan (**SADABS**; Bruker, 2005 ▶) *T*_min_ = 0.933, *T*_max_ = 0.965
18373 measured reflections
4882 independent reflections
3915 reflections with *I* > 2σ(*I*)
*R*_int_ = 0.030

#### Refinement

*R*[*F* ^2^ > 2σ(*F* ^2^)] = 0.045
*wR*(*F* ^2^) = 0.111
*S* = 1.04
4882 reflections
289 parameters
All H-atom parameters refined
Δρ_max_ = 0.47 e Å^−3^
Δρ_min_ = −0.29 e Å^−3^

### 

Data collection: *APEX2* (Bruker, 2005 ▶); cell refinement: *SAINT* (Bruker, 2005 ▶); data reduction: *SAINT*; program(s) used to solve structure: *SHELXTL* (Sheldrick, 2008 ▶); program(s) used to refine structure: *SHELXTL*; molecular graphics: *SHELXTL*; software used to prepare material for publication: *SHELXTL* and *PLATON* (Spek, 2009 ▶).

## Supplementary Material

Crystal structure: contains datablocks global, I. DOI: 10.1107/S1600536809050326/tk2581sup1.cif

Structure factors: contains datablocks I. DOI: 10.1107/S1600536809050326/tk2581Isup2.hkl

Additional supplementary materials:  crystallographic information; 3D view; checkCIF report

## Figures and Tables

**Table 1:** Hydrogen-bond geometry (Å, °)

*D*—H⋯*A*	*D*—H	H⋯*A*	*D*⋯*A*	*D*—H⋯*A*
C3—H3⋯O1^i^	0.962 (19)	2.39 (2)	3.225 (2)	145.6 (17)

Symmetry code: (i) 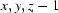.

**Fig. 1.**
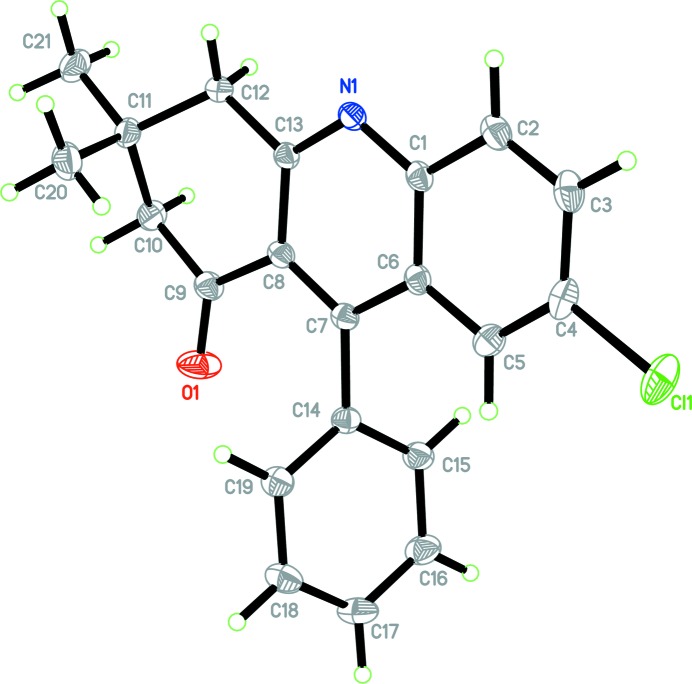
The molecular structure of the title compound, showing 50% probability displacement ellipsoids and the atom-numbering scheme.

**Fig. 2.**
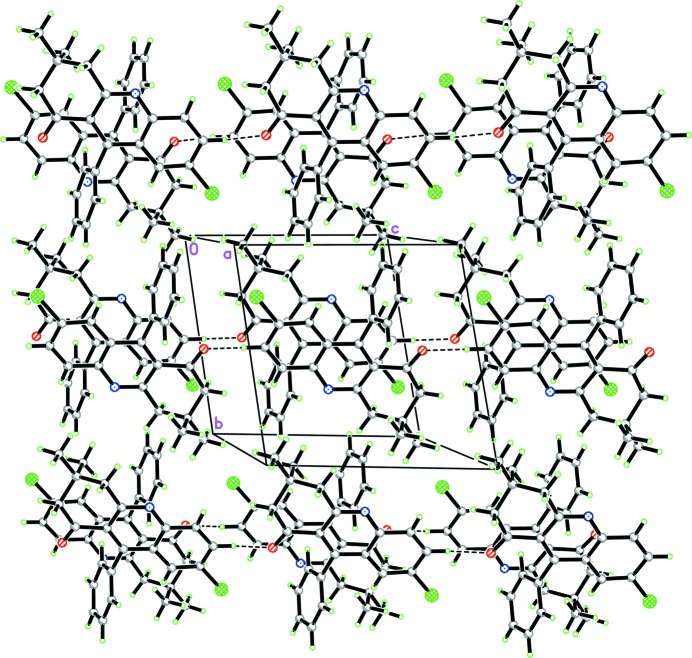
The crystal packing of the title compound, viewed approximately along the a axis, showing extended one-dimensional chains. The intermolecular interactions are shown as dashed lines.

## supplementary crystallographic information

### Comment

Quinolines and their derivatives are very important compounds because of their wide occurrence in natural products (Morimoto *et al.*, 1991; Michael, 1997) and biologically active compounds (Markees *et al.*, 1970; Campbell *et al.*, 1988). A large variety of quinolines have interesting physiological activities and have found attractive applications as pharmaceuticals, agrochemicals and as synthetic building blocks (Maguire *et al.*, 1994; Kalluraya & Sreenivasa, 1998; Roma *et al.*, 2000; Chen *et al.*, 2001). Because of their great importance, the synthesis of new derivatives of quinoline remains an active research area. Recently, we have reported the synthesis of some novel quinoline derivatives (Fun, Loh *et al.*, 2009; Fun, Yeap *et al.*, 2009).

In the title compound (Fig. 1), the quinoline ring system (C1–C8/C13/N1) is approximately planar with a maximum deviation of 0.035 (2) Å at atom C13. The mean plane through the quinoline ring forms a dihedral angle of 71.42 (6)° with the phenyl ring (C14–C19). The cyclohexanone (C8–C13) ring exists in a half-boat conformation. The puckering parameters (Cremer & Pople, 1975) are Q = 0.5017 (17) Å; Θ = 126.65 (18)° and φ = 352.8 (2)°. Bond lengths and angles are comparable to that in a closely related structure (Loh *et al.*, 2009).

In the crystal packing (Fig. 2), C3—H3···O1 (Table 1) hydrogen bonds link neighbouring molecules, forming extended one-dimensional chains along *c* axis.

### Experimental

A 1:1 mixture of 2-amino-5-chlorobenzophenone (0.2 g, 0.001 *M*), 5,5-dimethyl-1,3-cyclohexanedione (0.14 g, 0.001 *M*), and 1.0 ml concentrated HCl in distilled ethanol was irradiated for about 12 min under microwave irradiation at 240 W in a domestic microwave oven. The resulting mixture was poured on to ice and neutralized. The solid that formed was filtered, dried and purified by column chromatography using a 1:1 mixture of chloroform and petroleum ether. *M. pt.*: 459–461 K, yield: 45%.

### Refinement

All hydrogen atoms were located from the difference Fourier map and were refined freely [range of C–H = 0.936 (19) to 1.020 (2) Å].

### Crystal data

**Table:**

C_21_H_18_ClNO	*Z* = 2
*M**_r_* = 335.81	*F*(000) = 352
Triclinic, *P*1	*D*_x_ = 1.322 Mg m^−^^3^
Hall symbol: -P 1	Mo *K*α radiation, λ = 0.71073 Å
*a* = 9.8375 (1) Å	Cell parameters from 6537 reflections
*b* = 10.0525 (1) Å	θ = 2.3–32.2°
*c* = 10.1076 (1) Å	µ = 0.23 mm^−^^1^
α = 79.162 (1)°	*T* = 100 K
β = 63.389 (1)°	Block, colourless
γ = 70.928 (1)°	0.30 × 0.20 × 0.15 mm
*V* = 843.59 (2) Å^3^	

### Data collection

**Table:**

Bruker SMART APEXII CCD area-detector diffractometer	4882 independent reflections
Radiation source: fine-focus sealed tube	3915 reflections with *I* > 2σ(*I*)
graphite	*R*_int_ = 0.030
φ and ω scans	θ_max_ = 30.0°, θ_min_ = 2.2°
Absorption correction: multi-scan (*SADABS*; Bruker, 2005)	*h* = −13→13
*T*_min_ = 0.933, *T*_max_ = 0.965	*k* = −14→14
18373 measured reflections	*l* = −14→13

### Refinement

**Table:**

Refinement on *F*^2^	Primary atom site location: structure-invariant direct methods
Least-squares matrix: full	Secondary atom site location: difference Fourier map
*R*[*F*^2^ > 2σ(*F*^2^)] = 0.045	Hydrogen site location: inferred from neighbouring sites
*wR*(*F*^2^) = 0.111	All H-atom parameters refined
*S* = 1.04	*w* = 1/[σ^2^(*F*_o_^2^) + (0.0441*P*)^2^ + 0.4367*P*] where *P* = (*F*_o_^2^ + 2*F*_c_^2^)/3
4882 reflections	(Δ/σ)_max_ = 0.001
289 parameters	Δρ_max_ = 0.47 e Å^−^^3^
0 restraints	Δρ_min_ = −0.29 e Å^−^^3^

### Special details

**Table:**

Experimental. The crystal was placed in the cold stream of an Oxford Cyrosystems Cobra open-flow nitrogen cryostat (Cosier & Glazer, 1986) operating at 100.0 (1) K.
Geometry. All e.s.d.'s (except the e.s.d. in the dihedral angle between two l.s. planes) are estimated using the full covariance matrix. The cell e.s.d.'s are taken into account individually in the estimation of e.s.d.'s in distances, angles and torsion angles; correlations between e.s.d.'s in cell parameters are only used when they are defined by crystal symmetry. An approximate (isotropic) treatment of cell e.s.d.'s is used for estimating e.s.d.'s involving l.s. planes.
Refinement. Refinement of *F*^2^ against ALL reflections. The weighted *R*-factor *wR* and goodness of fit *S* are based on *F*^2^, conventional *R*-factors *R* are based on *F*, with *F* set to zero for negative *F*^2^. The threshold expression of *F*^2^ > σ(*F*^2^) is used only for calculating *R*-factors(gt) *etc*. and is not relevant to the choice of reflections for refinement. *R*-factors based on *F*^2^ are statistically about twice as large as those based on *F*, and *R*- factors based on ALL data will be even larger.

### Fractional atomic coordinates and isotropic or equivalent isotropic displacement parameters (Å^2^)

**Table:**

	*x*	*y*	*z*	*U*_iso_*/*U*_eq_	
Cl1	1.00497 (5)	0.24082 (4)	0.06504 (4)	0.03117 (11)	
O1	0.66039 (14)	0.51264 (12)	0.86939 (12)	0.0328 (3)	
N1	0.55017 (13)	0.70933 (12)	0.44602 (12)	0.0164 (2)	
C1	0.65323 (15)	0.59559 (14)	0.36408 (14)	0.0165 (3)	
C2	0.67188 (17)	0.59806 (16)	0.21588 (15)	0.0205 (3)	
C3	0.77851 (18)	0.49116 (16)	0.12519 (16)	0.0230 (3)	
C4	0.87017 (17)	0.37689 (15)	0.18098 (15)	0.0214 (3)	
C5	0.85517 (16)	0.36841 (15)	0.32311 (15)	0.0194 (3)	
C6	0.74409 (15)	0.47799 (14)	0.41947 (14)	0.0159 (3)	
C7	0.72349 (15)	0.47749 (14)	0.56874 (14)	0.0152 (2)	
C8	0.61798 (15)	0.59343 (14)	0.65057 (14)	0.0157 (2)	
C9	0.58812 (16)	0.60312 (15)	0.80857 (15)	0.0190 (3)	
C10	0.46337 (18)	0.73000 (15)	0.89036 (15)	0.0214 (3)	
C11	0.45479 (17)	0.86542 (14)	0.79119 (15)	0.0192 (3)	
C12	0.41995 (16)	0.83686 (14)	0.66761 (15)	0.0177 (3)	
C13	0.53443 (15)	0.70831 (14)	0.58292 (14)	0.0150 (2)	
C14	0.81958 (15)	0.35147 (14)	0.62420 (14)	0.0163 (3)	
C15	0.78844 (16)	0.22131 (15)	0.64521 (16)	0.0203 (3)	
C16	0.88422 (18)	0.10146 (16)	0.68699 (18)	0.0250 (3)	
C17	1.01186 (18)	0.11037 (16)	0.70617 (18)	0.0260 (3)	
C18	1.04371 (17)	0.23964 (16)	0.68430 (17)	0.0239 (3)	
C19	0.94831 (16)	0.35960 (15)	0.64384 (15)	0.0198 (3)	
C20	0.6109 (2)	0.90496 (18)	0.72693 (19)	0.0273 (3)	
C21	0.3203 (2)	0.98557 (16)	0.88205 (18)	0.0274 (3)	
H12B	0.4182 (19)	0.9185 (18)	0.5958 (19)	0.020 (4)*	
H12A	0.313 (2)	0.8231 (17)	0.7114 (18)	0.020 (4)*	
H5	0.920 (2)	0.2884 (19)	0.3566 (19)	0.027 (5)*	
H19	0.967 (2)	0.4527 (18)	0.6301 (19)	0.022 (4)*	
H3	0.791 (2)	0.4926 (19)	0.025 (2)	0.032 (5)*	
H15	0.702 (2)	0.2166 (18)	0.6296 (19)	0.026 (4)*	
H20C	0.700 (2)	0.8310 (18)	0.664 (2)	0.025 (4)*	
H17	1.077 (2)	0.0280 (19)	0.733 (2)	0.030 (5)*	
H18	1.130 (2)	0.2447 (19)	0.698 (2)	0.029 (5)*	
H21C	0.312 (2)	1.073 (2)	0.819 (2)	0.031 (5)*	
H10B	0.484 (2)	0.7408 (19)	0.974 (2)	0.034 (5)*	
H2	0.608 (2)	0.681 (2)	0.183 (2)	0.033 (5)*	
H10A	0.361 (2)	0.7063 (18)	0.933 (2)	0.026 (4)*	
H16	0.863 (2)	0.011 (2)	0.703 (2)	0.030 (5)*	
H21B	0.215 (2)	0.962 (2)	0.928 (2)	0.035 (5)*	
H21A	0.342 (2)	1.006 (2)	0.963 (2)	0.037 (5)*	
H20B	0.635 (2)	0.918 (2)	0.807 (2)	0.040 (5)*	
H20A	0.602 (2)	0.994 (2)	0.671 (2)	0.039 (5)*	

### Atomic displacement parameters (Å^2^)

**Table:**

	*U*^11^	*U*^22^	*U*^33^	*U*^12^	*U*^13^	*U*^23^
Cl1	0.0372 (2)	0.0252 (2)	0.02082 (18)	−0.00849 (15)	−0.00046 (15)	−0.00871 (14)
O1	0.0348 (6)	0.0349 (6)	0.0200 (5)	0.0068 (5)	−0.0154 (5)	−0.0010 (5)
N1	0.0181 (5)	0.0169 (5)	0.0169 (5)	−0.0055 (4)	−0.0098 (4)	0.0005 (4)
C1	0.0182 (6)	0.0181 (6)	0.0170 (6)	−0.0083 (5)	−0.0090 (5)	0.0006 (5)
C2	0.0250 (7)	0.0232 (7)	0.0181 (6)	−0.0102 (6)	−0.0115 (5)	0.0016 (5)
C3	0.0302 (7)	0.0268 (8)	0.0157 (6)	−0.0138 (6)	−0.0086 (6)	−0.0010 (5)
C4	0.0235 (7)	0.0195 (7)	0.0184 (6)	−0.0093 (5)	−0.0024 (5)	−0.0054 (5)
C5	0.0196 (6)	0.0173 (7)	0.0200 (6)	−0.0061 (5)	−0.0065 (5)	−0.0006 (5)
C6	0.0167 (6)	0.0167 (6)	0.0171 (6)	−0.0076 (5)	−0.0077 (5)	−0.0002 (5)
C7	0.0144 (6)	0.0158 (6)	0.0172 (6)	−0.0066 (5)	−0.0075 (5)	0.0019 (5)
C8	0.0169 (6)	0.0168 (6)	0.0154 (6)	−0.0058 (5)	−0.0084 (5)	0.0012 (5)
C9	0.0214 (6)	0.0206 (7)	0.0162 (6)	−0.0064 (5)	−0.0092 (5)	0.0013 (5)
C10	0.0282 (7)	0.0194 (7)	0.0157 (6)	−0.0044 (6)	−0.0097 (6)	−0.0014 (5)
C11	0.0256 (7)	0.0164 (6)	0.0179 (6)	−0.0060 (5)	−0.0105 (5)	−0.0015 (5)
C12	0.0202 (6)	0.0155 (6)	0.0191 (6)	−0.0033 (5)	−0.0109 (5)	−0.0003 (5)
C13	0.0160 (6)	0.0151 (6)	0.0165 (6)	−0.0058 (5)	−0.0082 (5)	0.0002 (5)
C14	0.0160 (6)	0.0165 (6)	0.0154 (6)	−0.0036 (5)	−0.0065 (5)	0.0002 (5)
C15	0.0186 (6)	0.0187 (7)	0.0249 (7)	−0.0053 (5)	−0.0107 (5)	0.0004 (5)
C16	0.0246 (7)	0.0164 (7)	0.0336 (8)	−0.0057 (6)	−0.0133 (6)	0.0026 (6)
C17	0.0211 (7)	0.0211 (7)	0.0324 (8)	−0.0024 (6)	−0.0131 (6)	0.0054 (6)
C18	0.0175 (6)	0.0270 (8)	0.0284 (7)	−0.0067 (6)	−0.0119 (6)	0.0035 (6)
C19	0.0195 (6)	0.0192 (7)	0.0211 (6)	−0.0071 (5)	−0.0089 (5)	0.0024 (5)
C20	0.0338 (8)	0.0263 (8)	0.0308 (8)	−0.0141 (7)	−0.0179 (7)	0.0006 (6)
C21	0.0376 (9)	0.0194 (7)	0.0233 (7)	−0.0019 (6)	−0.0134 (7)	−0.0056 (6)

### Geometric parameters (Å, °)

**Table:**

Cl1—C4	1.7402 (14)	C11—C12	1.5306 (19)
O1—C9	1.2122 (17)	C11—C20	1.532 (2)
N1—C13	1.3200 (16)	C12—C13	1.5075 (18)
N1—C1	1.3665 (17)	C12—H12B	0.989 (17)
C1—C6	1.4209 (18)	C12—H12A	0.987 (17)
C1—C2	1.4216 (18)	C14—C19	1.3960 (19)
C2—C3	1.367 (2)	C14—C15	1.3965 (19)
C2—H2	0.965 (19)	C15—C16	1.3931 (19)
C3—C4	1.409 (2)	C15—H15	0.944 (18)
C3—H3	0.966 (19)	C16—C17	1.386 (2)
C4—C5	1.366 (2)	C16—H16	0.969 (19)
C5—C6	1.4210 (19)	C17—C18	1.390 (2)
C5—H5	0.962 (18)	C17—H17	0.944 (18)
C6—C7	1.4288 (18)	C18—C19	1.3859 (19)
C7—C8	1.3866 (18)	C18—H18	0.936 (19)
C7—C14	1.4959 (17)	C19—H19	0.986 (17)
C8—C13	1.4363 (17)	C20—H20C	0.989 (18)
C8—C9	1.5052 (18)	C20—H20B	0.98 (2)
C9—C10	1.510 (2)	C20—H20A	0.97 (2)
C10—C11	1.5339 (19)	C21—H21C	0.987 (19)
C10—H10B	0.99 (2)	C21—H21B	1.02 (2)
C10—H10A	0.996 (18)	C21—H21A	1.00 (2)
C11—C21	1.529 (2)		
			
C13—N1—C1	118.00 (11)	C13—C12—C11	114.16 (11)
N1—C1—C6	122.92 (12)	C13—C12—H12B	108.2 (10)
N1—C1—C2	117.86 (12)	C11—C12—H12B	111.5 (10)
C6—C1—C2	119.21 (12)	C13—C12—H12A	107.6 (10)
C3—C2—C1	121.05 (13)	C11—C12—H12A	108.9 (10)
C3—C2—H2	123.0 (11)	H12B—C12—H12A	106.2 (13)
C1—C2—H2	115.9 (11)	N1—C13—C8	123.51 (12)
C2—C3—C4	118.94 (13)	N1—C13—C12	115.64 (11)
C2—C3—H3	121.0 (11)	C8—C13—C12	120.85 (11)
C4—C3—H3	120.0 (11)	C19—C14—C15	119.32 (12)
C5—C4—C3	122.32 (13)	C19—C14—C7	120.81 (12)
C5—C4—Cl1	119.04 (11)	C15—C14—C7	119.67 (12)
C3—C4—Cl1	118.64 (11)	C16—C15—C14	120.08 (13)
C4—C5—C6	119.59 (13)	C16—C15—H15	121.3 (11)
C4—C5—H5	119.5 (11)	C14—C15—H15	118.6 (11)
C6—C5—H5	120.9 (11)	C17—C16—C15	120.26 (14)
C1—C6—C5	118.86 (12)	C17—C16—H16	119.0 (11)
C1—C6—C7	118.33 (12)	C15—C16—H16	120.7 (11)
C5—C6—C7	122.77 (12)	C16—C17—C18	119.75 (13)
C8—C7—C6	118.01 (11)	C16—C17—H17	119.3 (11)
C8—C7—C14	124.97 (11)	C18—C17—H17	121.0 (11)
C6—C7—C14	117.02 (11)	C19—C18—C17	120.37 (14)
C7—C8—C13	119.21 (11)	C19—C18—H18	120.5 (11)
C7—C8—C9	122.07 (11)	C17—C18—H18	119.1 (11)
C13—C8—C9	118.72 (12)	C18—C19—C14	120.22 (13)
O1—C9—C8	121.74 (13)	C18—C19—H19	121.7 (10)
O1—C9—C10	120.60 (12)	C14—C19—H19	118.0 (10)
C8—C9—C10	117.65 (11)	C11—C20—H20C	111.5 (10)
C9—C10—C11	113.43 (12)	C11—C20—H20B	110.4 (12)
C9—C10—H10B	107.8 (11)	H20C—C20—H20B	108.1 (15)
C11—C10—H10B	111.5 (11)	C11—C20—H20A	110.4 (12)
C9—C10—H10A	106.2 (10)	H20C—C20—H20A	110.1 (16)
C11—C10—H10A	109.8 (10)	H20B—C20—H20A	106.1 (17)
H10B—C10—H10A	107.7 (15)	C11—C21—H21C	110.1 (11)
C21—C11—C12	109.62 (12)	C11—C21—H21B	111.3 (11)
C21—C11—C20	109.35 (12)	H21C—C21—H21B	108.7 (15)
C12—C11—C20	110.87 (12)	C11—C21—H21A	110.5 (11)
C21—C11—C10	109.44 (12)	H21C—C21—H21A	107.0 (15)
C12—C11—C10	106.87 (11)	H21B—C21—H21A	109.1 (16)
C20—C11—C10	110.65 (12)		
			
C13—N1—C1—C6	−0.51 (19)	C8—C9—C10—C11	−34.80 (18)
C13—N1—C1—C2	−178.97 (12)	C9—C10—C11—C21	177.24 (12)
N1—C1—C2—C3	176.78 (13)	C9—C10—C11—C12	58.62 (16)
C6—C1—C2—C3	−1.7 (2)	C9—C10—C11—C20	−62.19 (16)
C1—C2—C3—C4	0.3 (2)	C21—C11—C12—C13	−172.63 (12)
C2—C3—C4—C5	0.7 (2)	C20—C11—C12—C13	66.54 (15)
C2—C3—C4—Cl1	179.70 (11)	C10—C11—C12—C13	−54.13 (15)
C3—C4—C5—C6	−0.2 (2)	C1—N1—C13—C8	−0.93 (19)
Cl1—C4—C5—C6	−179.21 (10)	C1—N1—C13—C12	179.64 (11)
N1—C1—C6—C5	−176.26 (12)	C7—C8—C13—N1	1.2 (2)
C2—C1—C6—C5	2.18 (19)	C9—C8—C13—N1	−178.91 (12)
N1—C1—C6—C7	1.61 (19)	C7—C8—C13—C12	−179.39 (12)
C2—C1—C6—C7	−179.94 (12)	C9—C8—C13—C12	0.50 (18)
C4—C5—C6—C1	−1.2 (2)	C11—C12—C13—N1	−154.25 (12)
C4—C5—C6—C7	−179.02 (13)	C11—C12—C13—C8	26.30 (18)
C1—C6—C7—C8	−1.27 (18)	C8—C7—C14—C19	−72.90 (18)
C5—C6—C7—C8	176.52 (12)	C6—C7—C14—C19	106.43 (15)
C1—C6—C7—C14	179.35 (11)	C8—C7—C14—C15	112.34 (15)
C5—C6—C7—C14	−2.86 (19)	C6—C7—C14—C15	−68.33 (16)
C6—C7—C8—C13	−0.03 (18)	C19—C14—C15—C16	0.7 (2)
C14—C7—C8—C13	179.29 (12)	C7—C14—C15—C16	175.57 (13)
C6—C7—C8—C9	−179.91 (12)	C14—C15—C16—C17	−0.8 (2)
C14—C7—C8—C9	−0.6 (2)	C15—C16—C17—C18	0.3 (2)
C7—C8—C9—O1	3.4 (2)	C16—C17—C18—C19	0.2 (2)
C13—C8—C9—O1	−176.50 (14)	C17—C18—C19—C14	−0.2 (2)
C7—C8—C9—C10	−176.29 (13)	C15—C14—C19—C18	−0.2 (2)
C13—C8—C9—C10	3.83 (18)	C7—C14—C19—C18	−175.01 (13)
O1—C9—C10—C11	145.52 (14)		

### Hydrogen-bond geometry (Å, °)

**Table:**

*D*—H···*A*	*D*—H	H···*A*	*D*···*A*	*D*—H···*A*
C3—H3···O1^i^	0.962 (19)	2.39 (2)	3.225 (2)	145.6 (17)

Symmetry codes: (i) *x*, *y*, *z*−1.

## References

[bb1] Bruker (2005). *APEX2*, *SAINT* and *SADABS*. Bruker AXS Inc., Madison, Wisconsin, USA.

[bb2] Campbell, S. F., Hardstone, J. D. & Palmer, M. J. (1988). *J. Med. Chem.* **31**, 1031–1035. 10.1021/jm00400a025 2896245

[bb3] Chen, Y.-L., Fang, K.-C., Sheu, J.-Y., Hsu, S.-L. & Tzeng, C.-C. (2001). *J. Med. Chem.* **44**, 2374–2377. 10.1021/jm0100335 11428933

[bb4] Cosier, J. & Glazer, A. M. (1986). *J. Appl. Cryst.* **19**, 105–107.

[bb5] Cremer, D. & Pople, J. A. (1975). *J. Am. Chem. Soc.* **97**, 1354–1358.

[bb6] Fun, H.-K., Loh, W.-S., Sarveswari, S., Vijayakumar, V. & Reddy, B. P. (2009). *Acta Cryst.* E**65**, o2688–o2689. 10.1107/S1600536809040306 PMC2971397 21578294

[bb7] Fun, H.-K., Yeap, C. S., Sarveswari, S., Vijayakumar, V. & Prasath, R. (2009). *Acta Cryst.* E**65**, o2665–o2666. 10.1107/S1600536809040252 PMC2971305 21578276

[bb8] Kalluraya, B. & Sreenivasa, S. (1998). *Farmaco*, **53**, 399–404. 10.1016/s0014-827x(98)00037-8 9764472

[bb9] Loh, W.-S., Fun, H.-K., Sarveswari, S., Vijayakumar, V. & Reddy, B. P. (2009). *Acta Cryst.* E**65**, o3144–o3145. 10.1107/S1600536809048934 PMC2971847 21578864

[bb10] Maguire, M. P., Sheets, K. R., McVety, K., Spada, A. P. & Zilberstein, A. (1994). *J. Med. Chem.* **37**, 2129–2137. 10.1021/jm00040a003 8035419

[bb11] Markees, D. G., Dewey, V. C. & Kidder, G. W. (1970). *J. Med. Chem.* **13**, 324–326. 10.1021/jm00296a048 5418519

[bb12] Michael, J. P. (1997). *Nat. Prod. Rep.* **14**, 605–608.

[bb13] Morimoto, Y., Matsuda, F. & Shirahama, H. (1991). *Synlett*, **3**, 202–203.

[bb14] Roma, G., Braccio, M. D., Grossi, G., Mattioli, F. & Ghia, M. (2000). *Eur. J. Med. Chem.* **35**, 1021–1026. 10.1016/s0223-5234(00)01175-2 11137230

[bb15] Sheldrick, G. M. (2008). *Acta Cryst.* A**64**, 112–122. 10.1107/S0108767307043930 18156677

[bb16] Spek, A. L. (2009). *Acta Cryst.* D**65**, 148–155. 10.1107/S090744490804362X PMC2631630 19171970

